# Modeling Herpes Simplex Virus 1 Infections in Human Central Nervous System Neuronal Cells Using Two- and Three-Dimensional Cultures Derived from Induced Pluripotent Stem Cells

**DOI:** 10.1128/JVI.00111-19

**Published:** 2019-04-17

**Authors:** Leonardo D’Aiuto, David C. Bloom, Jennifer N. Naciri, Adam Smith, Terri G. Edwards, Lora McClain, Jason A. Callio, Morgan Jessup, Joel Wood, Kodavali Chowdari, Matthew Demers, Eric E. Abrahamson, Milos D. Ikonomovic, Luigi Viggiano, Roberta De Zio, Simon Watkins, Paul R. Kinchington, Vishwajit L. Nimgaonkar

**Affiliations:** a Department of Psychiatry, University of Pittsburgh School of Medicine Western Psychiatric Institute and Clinic, Pittsburgh, Pennsylvania, USA; b Department of Molecular Genetics & Microbiology, University of Florida College of Medicine, Gainesville, Florida, USA; c Magee-Women’s Research Institute, Pittsburgh, Pennsylvania, USA; d Department of Pathology, Division of Neuropathology, University of Pittsburgh School of Medicine, Pittsburgh, Pennsylvania, USA; e Department of Cell Biology, University of Pittsburgh, Pittsburgh, Pennsylvania, USA; f Department of Neurology, University of Pittsburgh School of Medicine, Pittsburgh, Pennsylvania, USA; g Department of Biology, University of Bari Aldo Moro, Bari, Italy; h Dipartimento di Bioscienze, Biotecnologie e Biofarmaceutica, Università degli Studi di Bari, Bari, Italy; i Department of Ophthalmology, University of Pittsburgh School of Medicine, Pittsburgh, Pennsylvania, USA; University of California, Irvine

**Keywords:** herpes simplex virus 1 (HSV-1), human induced pluripotent stem cells (hiPSCs), three-dimensional (3D) neuronal cultures, neurodegeneration, organoid

## Abstract

Herpes simplex virus 1 (HSV-1) establishes latency in both peripheral nerve ganglia and the central nervous system (CNS). The outcomes of acute and latent infections in these different anatomic sites appear to be distinct. It is becoming clear that many of the existing culture models using animal primary neurons to investigate HSV-1 infection of the CNS are limited and not ideal, and most do not recapitulate features of CNS neurons. Human induced pluripotent stem cells (hiPSCs) and neurons derived from them are documented as tools to study aspects of neuropathogenesis, but few have focused on modeling infections of the CNS. Here, we characterize functional two-dimensional (2D) CNS-like neuron cultures and three-dimensional (3D) brain organoids made from hiPSCs to model HSV-1–human–CNS interactions. Our results show that (i) hiPSC-derived CNS neurons are permissive for HSV-1 infection; (ii) a quiescent state exhibiting key landmarks of HSV-1 latency described in animal models can be established in hiPSC-derived CNS neurons; (iii) the complex laminar structure of the organoids can be efficiently infected with HSV, with virus being transported from the periphery to the central layers of the organoid; and (iv) the organoids support reactivation of HSV-1, albeit less efficiently than 2D cultures. Collectively, our results indicate that hiPSC-derived neuronal platforms, especially 3D organoids, offer an extraordinary opportunity for modeling the interaction of HSV-1 with the complex cellular and architectural structure of the human CNS.

**IMPORTANCE** This study employed human induced pluripotent stem cells (hiPSCs) to model acute and latent HSV-1 infections in two-dimensional (2D) and three-dimensional (3D) CNS neuronal cultures. We successfully established acute HSV-1 infections and infections showing features of latency. HSV-1 infection of the 3D organoids was able to spread from the outer surface of the organoid and was transported to the interior lamina, providing a model to study HSV-1 trafficking through complex neuronal tissue structures. HSV-1 could be reactivated in both culture systems; though, in contrast to 2D cultures, it appeared to be more difficult to reactivate HSV-1 in 3D cultures, potentially paralleling the low efficiency of HSV-1 reactivation in the CNS of animal models. The reactivation events were accompanied by dramatic neuronal morphological changes and cell-cell fusion. Together, our results provide substantive evidence of the suitability of hiPSC-based neuronal platforms to model HSV-1–CNS interactions in a human context.

## Abstract (precis)

This study employed human induced pluripotent stem cells (hiPSCs) to model acute and latent HSV-1 infections in two-dimensional (2D) and three-dimensional (3D) CNS neuronal cultures. We successfully established acute HSV-1 infections and infections showing features of latency. HSV-1 infection of the 3D organoids was able to spread from the outer surface of the organoid and was transported to the interior lamina, providing a model to study HSV-1 trafficking through complex neuronal tissue structures. HSV-1 could be reactivated in both culture systems; though, in contrast to 2D cultures, it appeared to be more difficult to reactivate HSV-1 in 3D cultures, potentially paralleling the low efficiency of HSV-1 reactivation in the CNS of animal models. The reactivation events were accompanied by dramatic neuronal morphological changes and cell-cell fusion. Together, our results provide substantive evidence of the suitability of hiPSC-based neuronal platforms to model HSV-1–CNS interactions in a human context.

## INTRODUCTION

Human herpes simplex virus 1 (HSV-1) infection is a highly prevalent, human-specific infection that causes substantial morbidity, including recurrent cold sores, ocular disease that can lead to blindness, and a rare but devastating encephalitis. Current understanding of the mechanisms regulating HSV-1 lytic infections, latency, reactivation, and neuropathogenesis comes primarily from small-animal models, such as mice and rabbits. While these models are instructive, there is a critical unmet need for human models, particularly given that HSV-1 is a human species-specific virus that has evolved over millennia by interacting with human cells. Indeed, it has become clear that there are often critical differences between the HSV-1 behaviors seen in animal models and the pathogenesis seen in human disease, suggesting that such species-specific interactions are critical in resolving a mechanistic understanding of the processes of infection. In particular, while HSV-1 latency mechanisms have been well studied in animal systems, latency remains a poorly understood process that involves interactions between the virus and its host cell. HSV-1 latency has been defined as the reversible retention of a quiescent yet functional viral genome in neuronal nuclei with no apparent production of infectious virions and the transcriptional repression of most viral genes, perhaps with the exception of the expression of a latency-associated transcript (LAT) and its spliced products and microRNAs (miRNAs) in some latently infected neurons (1, 2). This descriptive definition is perhaps quite debatable. In fact, single-cell gene expression analysis of latently infected mouse neurons has indicated that (i) more than 50% of latently infected neurons express more than one viral lytic gene at any one time (3); (ii) the presence of HSV-1, even in a quiescent state, alters the host neuronal transcriptional circuits (3); and (iii) not every latently infected neuron expresses the LAT products. Studies in animal models indicated that during latency, the HSV-1 genome is an episome highly enriched in heterochromatin repressive marks H3K9me3 and H3K27me3 (4, 5). The region encoding the LAT is the only viral region enriched in epigenetic marks typical of transcriptionally active regions, such as H3K4me2, H3K9ac, and H3K14ac, during latency (6). Evidence suggests that this region is also insulated by CTCF marks that serve to prevent transcriptional silencing (6). Thus, a more plausible dynamic model of latency suggests that the lack of productive infection during latency results from an inhibitory host response opposing HSV-1 reactivation, rather than an inactive viral state. The absence of an adequate human cellular model and the limited availability of primary human neuronal cultures have hindered progress to investigate HSV-1 latency, the chromatin organization of HSV-1 genomes, and the transcription of viral miRNAs in human neurons.

These difficulties have also impeded models of the interactions of HSV-1 with the human central nervous system (CNS). HSV-1 infection of the CNS is the most common cause of encephalitis (herpes simplex encephalitis [HSE]), which has an incidence of 2 to 4/100,000 people annually, and is associated with rising rates of encephalitis in children (7–9). Even though antiviral therapy by acyclovir derivatives has significantly reduced the mortality to approximately 25%, patients who survive HSE often experience significant long-term neurological sequelae (10). Thus, human *in vitro* systems are critically needed to investigate HSV-1 genetics and epigenetics, to model HSV-1 infection of the human CNS, and to advance our understanding of the molecular mechanisms involved in HSV-1 latency and reactivation. Such models would facilitate the development of more efficacious and long-lasting therapies for prophylaxis and treatment of HSV-1 infections, with a goal of improving the neurological sequelae in encephalitis survivors.

The experimental approaches to model the infection of neurotropic viruses have changed profoundly with the advent of human induced pluripotent stem cell (hiPSC) technologies, which allow the generation and manipulation of potentially limitless numbers of live human hiPSC-derived neuronal lineage cells reprogrammed from specific individuals. Thus, hiPSC-based models offer the potential to investigate multiple aspects of the pathogenesis of neurotropic viruses at the cellular and molecular levels (11–14). To more accurately model the host-pathogen interaction, recent advances in stem cell differentiation strategies allow for the generation of three-dimensional (3D) neuron cultures, referred to as “brain organoids,” that recapitulate features of a developing brain, including neuronal heterogeneity as well as a complex lamina-like architecture (15, 16). In this study, we utilized hiPSC-derived two-dimensional (2D) and 3D neuronal models to investigate HSV-1 infection. Our goal was not to compare the 2D and 3D models; we attempted to recapitulate CNS infection with HSV-1 and to investigate different facets of infection.

## RESULTS

### hiPSC-derived CNS neurons are permissive to HSV-1 infection in 2D cultures.

**FIG 1:**
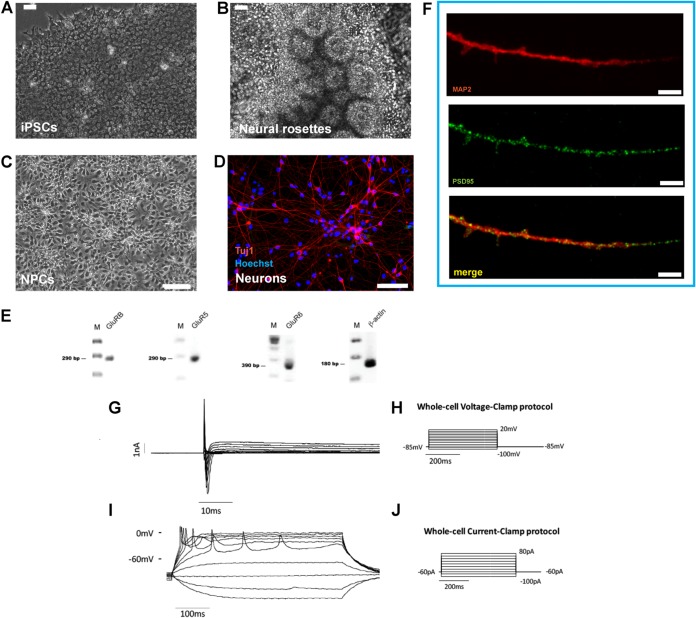
Neuronal differentiation of human iPSCs (hiPSCs) in 2D cultures. (A to F) hiPSCs (A) are differentiated into columnar epithelial cells, forming neural rosettes (B). (C) hiPSC-derived neural rosettes are expanded as monolayer cultures of neural stem cells/neural progenitor cells (collectively referred as neural precursor cells [NPCs] in this study). (D) NPCs are further differentiated into neurons, illustrated using Tuj1 immunofluorescence (red) with Hoechst 33342 counterstaining of nuclei (blue). (E) These cells express the glutamate receptors GluRB, GluR5, and GluR6. Lanes M, molecular size markers. (F) Coimmunostaining of hiPSC-derived neurons with PSD-95 (green) and MAP2 (red) revealed PSD-95-labeled dendritic protrusions resembling a spine. (A to C) Phase-contrast microscopy; (D, F) confocal fluorescence microscopy. Bars, 50 μm (A and B), 100 μm (C), 75 μm (D), 5 μm (F). (G to J) Electrophysiological recordings of hiPSC-derived neurons. In voltage clamp experiments on cells with a resting potential equal to or more negative than −40 mV, when the membrane potential was depolarized from −100 mV to 20 mV starting from a holding potential of −85 mV, two notable currents were evoked: a fast inward Na^+^ component (evoked starting from −30 mV until 20 mV, with a maximum value of current at −30 mV of −2,263 ± 329.4 pA; mean ± SEM, *n* = 4 cells) and a slower outward K^+^ component (evoked starting from −30 mV until 20 mV, with a maximum value of current at 20 mV of 1,020 ± 179.5 pA; mean ± SEM, *n* = 4 cells). In current clamp mode, depolarizing steps of current from −100 pA to 80 pA were used to detect the ability of the cells to generate action potentials. hiPSC-derived neurons showed repetitive evoked action potentials that were in line with the recorded currents. (G) Voltage clamp recording. The currents recorded consist of an inward Na^+^ component and of an outward K^+^ component. (H) Currents were recorded using a voltage clamp protocol consisting of depolarizing steps from −100 mV to 20 mV starting from a holding potential of −85 mV. (I and J) A representative trace of evoked action potentials obtained in current clamp mode (I) injecting depolarizing steps of current from −100 pA to 80 pA (J).

We recently reported the sensitivity of human 2D hiPSC-derived neuronal cultures to HSV-1 infection (11). These neurons exhibit features of dorsolateral prefrontal cortex pyramidal neurons (17). Also, these neurons express the UNC93B1 gene (TPM 19.7228), which plays a protective role in HSV-1 infection of the brain (18). In order to further study the interaction of HSV-1 with CNS neurons, we investigated the expression of the immediate early protein ICP4 in the nuclei of HSV-1 infected MAP2 (microtubule associated protein 2)-positive hiPSC-derived CNS neurons (referred to here as hiPSC-neurons), generated as previously described (17) (Fig. 1).

**FIG 2:**
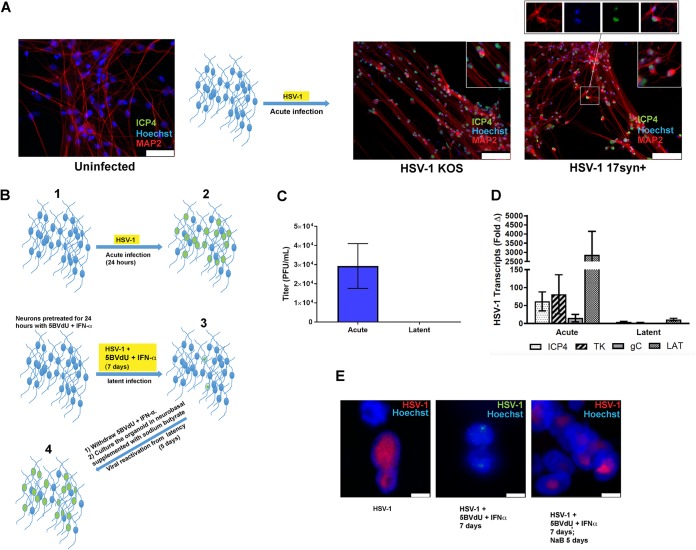
Establishment of latent HSV-1 infection in CNS neurons in 2D cultures. (A) Microphotographs depicting uninfected and HSV-1 acutely infected neurons (strains 17*syn*^+^ and KOS). Cells were processed using antibodies generated against ICP4 and MAP2 48 h after the infection. (Insets) Enlarged details. In particular, the inset at the top right shows that the HSV-1 antigen ICP4 is detected in the Hoechst-negative areas of infected neuronal nuclei. (B) Flow chart illustrating culture treatment paradigm: neurons are infected with HSV-1 (KOS) for 24 h (acute infection) (step 1), infected with HSV-1 and exposed to 5BVdU and IFN-α for 7 days (latent infection) (step 2), or infected with HSV-1 and cultured with 5BVdU and IFN-α for 7 days (step 3), after which the drugs are removed from the culture medium and infected cells are further cultured for 5 days in neurobasal medium with sodium butyrate (NaB) (step 4). (C) The viral titer in the supernatants from the acutely and latently infected culture plates was measured using a plaque assay on Vero cells. (D) A comparative analysis of viral gene expression in acutely and latently infected neurons demonstrates downregulation in latent infection. (E) Fluorescent *in situ* hybridization (FISH) was performed using the HSV-1 genome as a probe to detect viral DNA in acutely (left) and latently (middle) infected neurons, and during reactivation (right). Bars, 50 μm in uninfected neurons (A), 100 μm in neurons infected with HSV-1 KOS and 17*syn*+ (A), and 5 μm (E).

hiPSC-neurons (derived from neural progenitor cells [NPCs] that were differentiated for 6 weeks) were infected with HSV-1 strains KOS and 17*syn*+ at a multiplicity of infection (MOI) approximately equaling 0.3. At 48 h postinfection (hpi), the expression of ICP4 was investigated by immunocytochemistry. Cultures showed the localization of ICP4 to nuclei of MAP2-immunolabeled neurons in HSV-1-infected cultures (Fig. 2A). The ICP4 protein was found to be distributed in Hoechst-negative regions of the infected nuclei, while the host chromatin (Hoechst positive) was mainly concentrated at the nuclear periphery (Fig. 2A). This distribution pattern of ICP4 in relation to the host chromatin has been seen by others and concluded to reflect HSV-1 active replication (19). These data support our earlier observation and suggest that hiPSC-neurons are permissive to HSV-1 lytic infections. Subsequent studies used this model to further define virus-host interactions, viral latency, and potential reactivation in human neurons.

### Analysis of chromatin composition and chromatin accessibility state of HSV-1 episomes at the promoters of viral lytic genes and LAT sequences in acutely and latently infected hiPSC-neurons.

We previously developed an hiPSC-based neuronal culture model of HSV-1 latency which was adapted from a system using human sensory neurons isolated from aborted fetuses (20). Here latent infections were established in hiPSC-neurons infected at an MOI of 0.3 in the presence of the antivirals (*E*)-5-(2-bromovinyl)-2′-deoxyuridine (5BVdU; 30 μM) and alpha interferon (IFN-α; 125 U/ml) for 7 days. Under these culture conditions, the defining features of HSV-1 latency were observed, in that we saw the downregulation of HSV-1 lytic genes, expression of the LAT region, and a large reduction of the viral DNA copy number compared to those seen during lytic infection. Viral genomes also localized to the nuclear periphery, which differed from their locations seen during lytic infection (11).

Withdrawal of 5BVdU and IFN-α for 5 days did not lead to HSV-1 reactivation, suggesting that the hiPSC-neurons in these cultures harbored a latent infection. This is similar to the findings for systems using murine and rat *ex vivo* primary neuron cultures (21). However, HSV-1 reactivation was consistently observed when the antivirals 5BVdU and IFN-α were withdrawn from the culture medium and the infected cells were treated for 5 days with a type I histone deacetylase (HDAC) inhibitor, sodium butyrate (NaB; 5 mM). This produced infectious virus detected by plaque assay, indicating that HSV-1 latency in hiPSC-neurons is reversible.

To further characterize our latency model (11), we analyzed the state of HSV-1 chromatin in infected hiPSC-neurons after infection with a genetically engineered HSV-1 isolate, based on the KOS strain, which expressed enhanced green fluorescent protein (EGFP) and monomeric red fluorescent protein (RFP) under the control of the viral promoters ICP0 and glycoprotein C (gC), respectively. hiPSC-neurons were first cultured in Matrigel-coated 6-well plates (2 × 10^6^ cells/well) and then acutely infected at an MOI of 0.3 or infected at the same MOI and incubated with antivirals 5BVdU and IFN-α to induce the latent state (Fig. 2B). To assess the gene expression profiles during acute infection, cells were harvested at 24 h after infection, while for latency, infected cells incubated with the antivirals 5BVdU and IFN-α were harvested at 7 days postinfection (p.i.) (Fig. 2B). Supernatants were assayed for infectious virus, while the cell pellets were processed for RNA extraction. While infectious virus was detectable in the supernatant of acutely infected cultures, it was never detected in the supernatant of latently infected cultures (Fig. 2C). Reverse transcription (RT)-quantitative PCR (qPCR) showed a drastic reduction of the expression of the viral genes for ICP4, thymidine kinase (TK), and gC, with the gene for gC not being detectable at 7 days p.i. (Fig. 2D). High levels of the LAT transcript, which likely reflects the LAT primary transcript that accumulates abundantly at late times as a gamma transcript in productive infection of most cell types (22), were detected at 24 h postinfection. At 7 days p.i., LAT was decreased in expression compared to its level of expression at the 24-h time point (Fig. 2D). When we performed three-dimensional fluorescence *in situ* hybridization (3D-FISH) using probes from the viral genome, numerous hybridizing signals were observed in the nuclei of acutely infected neurons as large areas of nuclear positivity (Fig. 2E), consistent with the viral replication factories seen earlier. Conversely, in latently infected neurons, only a few weak hybridization signals were detected as small puncta (Fig. 2E). After the exposure of latently infected neurons to NaB to induce viral reactivation for 5 days, there was an abundance of hybridizing signals showing diverse nuclear distributions, which was comparable to the findings seen in acutely infected cells. These results indicate a reactivation from latency and subsequent DNA replication (Fig. 2E).

**FIG 3:**
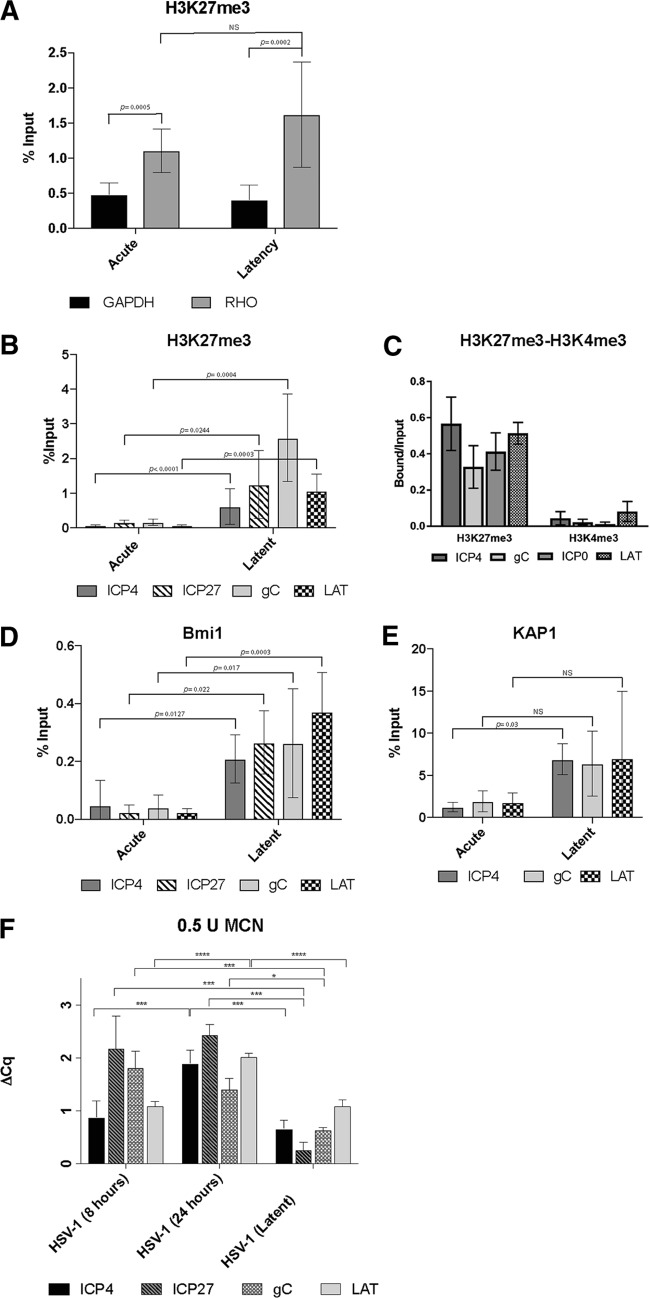
Chromatin analysis of HSV-1 in acutely and latently infected hiPSC-derived CNS neurons. (A) Validation of the ChIPs using anti-H3K27me3 by analyzing the results of ChIP-qPCR with positive-control PCR primers/probe to rhodopsin (*RHO*) compared to those of ChIP-qPCR with a negative-control PCR primer/probe to *GAPDH*. (B to E) Comparison of the enrichment of H3K27me3 (B and C), H3K4me3 (C), the polycomb group protein Bmi1 (D), and the coregulator of the Krüppel-associated box-containing zinc finger proteins (KAP1) (E) at the indicated viral promoter regions in acutely and latently infected hiPSC-derived neuronal cultures via ChIP analysis. The data represent averages from three independent experiments. The ChIP-qPCR data in panels A, B, D, and E were normalized using the percent input method, while in panel C, the relative quantities of enrichment are represented as bound/unbound. Error bars represent standard deviations (SD). *P* values were determined using Student's t test. (F) Analysis of HSV-1 chromatin accessibility to MCN by ChART-PCR. hiPSC-neurons were acutely infected with an HSV-1 construct expressing reporter genes EGFP and RFP under the control of viral promoters for 8 h and 24 h and latently infected with HSV-1 for 7 days at an MOI of 0.3. Following MCN digestion and DNA extraction, real-time qPCR was conducted using primers specific to promoter regions of viral genes. MCN accessibility was determined by calculating the difference in the amounts of undigested DNA and digested DNA (Δ*C_q_*). Error bars represent the standard deviation of the Δ*C_q_* values. *P* values were determined using a *post hoc* Tukey test. *, *P* ≤ 0.05; ***, *P* ≤ 0.001; ****, *P* < 0.0001; NS, not significant.

Next, we analyzed the association of the repressive histone mark H3K27me3 associated with the promoter region of the HSV-1 genes for ICP4, ICP27, and gC and the LAT locus using chromatin immunoprecipitation (ChIP) of lytic and latently infected neurons. Chromatin was prepared from acutely and latently infected cells. The relative quantities of immunoprecipitated DNAs were analyzed by real-time quantitative PCR and normalized using the percent input method. The efficiency of the immunoprecipitation was assessed by comparing the enrichment of H3K27me3 at the promoter region of rhodopsin (*RHO*), a known transcriptionally silent locus in neuronal cells and at the housekeeping gene *GAPDH* (the gene for glyceraldehyde-3-phosphate dehydrogenase). A 2.3- and 4-fold enrichment of H3K27me3 at the *RHO* locus relative to the level at *GAPDH* was observed in acutely and latently infected cultures, respectively (*P* = 0.0005 and *P* = 0.0002, respectively) (Fig. 3A). The difference in H3K27me3 enrichment at the *RHO* promoter region between acutely and latently infected cultures was not statistically significant (*P* = 0.11) (Fig. 3A). ChIP analysis of the repressive and permissive respective marks H3K4me3 and H3K27me3 at the ICP0, ICP4 gC, and LAT promoters revealed a 50- to 100-fold increase in H3K27me3 and a corresponding loss of the H3K4me3 marker at these loci (Fig. 3B and C). These results indicate that the transcriptionally repressive and heterochromatin mark H3K27me3 is associated with lytic genes during latency in the hiPSC-neurons.

Given this, we subsequently investigated the recruitment of human neuronal heterochromatin factors to the HSV-1 genomes, including the polycomb group protein Bmi1 (5), heterochromatin protein 1 (HP1) (23), and KAP1 (a coregulator of the Krüppel-associated box-containing zinc finger proteins [KRAB-ZFPs]) (24). A modest but significant enrichment (a 4.45- to 16.15-fold increase; *P* < 0.05) of the polycomb group protein Bmi1 was observed at the promoter regions of the aforementioned HSV-1 genes in latently infected cells compared to its level in acutely infected cells (Fig. 3D). A significant enrichment of KAP1 (a 5.65-fold increase) was observed only at the ICP4 promoter region in latently infected cells (Fig. 3E), although the enrichment at the other promoter regions tested strongly trended in the same manner. This suggests a possible involvement of the Krüppel-associated box-containing zinc finger proteins in the silencing of HSV-1 genes during the establishment and/or the maintenance of latency. No enrichment of HP1 at the aforementioned HSV-1 promoter regions was observed in latently infected cells (data not shown). Taken together, the enrichment of H3K27me3 and Bmi1 and the reduction of H3K4me3 indicate that the HSV-1 genome acquires heterochromatic features in our latency model. Furthermore, our results indicate an involvement of KAP1 in the establishment and/or the maintenance of HSV-1 latency.

The accessibility to the micrococcal nuclease (MCN) of HSV-1 chromatin was analyzed utilizing a method termed chromatin accessibility RT-PCR (ChART-PCR) (25). Cells were infected using recombinant HSV-1 KOS expressing the reporter genes EGFP and RFP. Nuclei isolated from neurons that were acutely infected at 24 hpi were digested with increasing amounts of MCN (0.5 U, 1 U, 3 U, and 5 U) at 39°C for 20 min and compared to control nuclei that were not digested with MCN. After DNA purification, the extent of the digestion was analyzed by agarose gel electrophoresis. Five nanograms of undigested and partially MCN-digested DNA from each condition was used to perform TaqMan real-time PCR using EGFP-specific primer sequences. No significant difference in changes in quantification cycle (Δ*C_q_*) values was observed among the different conditions (*P* = 0.0930) (data not shown). We then evaluated nuclei isolated from acutely infected cells collected at 8 hpi and 24 hpi and from latently infected cells. These were digested with 0.5 U of MCN as described above alongside a control that was not digested with MCN. Genomic DNAs from undigested and digested samples were analyzed by TaqMan real-time PCR using primer pairs specific for HSV-1 genes ICP4, ICP27, gC, and LAT (Fig. 3F). One-way analysis of variance (ANOVA) was performed on the Δ*C_q_* values for each gene among the three groups (the 8-h, 24-h, and latent infection groups) and yielded significant findings in each group *F*_ICP4_(2,11) = 18.95, *P* = 0.0003; *F*_ICP27_(2,11) = 18.76, *P* = 0.0003; *F*_gC_(2,11) = 20.57, *P* = 0.0002; *F*_LAT_(2,10) = 65.16, (*P* < 0.0001).

A *post hoc* Tukey test for ICP4 showed group differences between the latent and 24-h infection groups and the 8-h and 24-h infection groups at *P* values of 0.0005 and 0.0005, respectively. A *post hoc* Tukey test for ICP27 showed group differences between the latent and 8-h infection groups and the latent and the 24-h infection groups at *P* values of 0.0004 and 0.0007, respectively. The 8-h infection group was not significantly different from the 24-h infection group (*P* = 0.7379). A *post hoc* Tukey test for gC showed group differences between the latent and 8-h infection groups and the latent and the 24-h infections groups at *P* values of 0.0001 and 0.0139, respectively. The 8-h infection group was not significantly different from the 24-h infection group (*P* = 0.1085). A *post hoc* Tukey test for LAT showed group differences between the latent and 24-h infection groups and the 8-h and the 24-h infection groups at *P* values of <0.0001 and <0.0001, respectively. These results provide additional evidence that HSV-1 genomes are organized into heterochromatin in our induced pluripotent stem cell (iPSC)-based latency model. Significant differences were observed at the ICP27 and gC promoter regions between cells acutely infected for 8 h and latently infected cells (*P* < 0.0004 and *P* = 0.0001, respectively) but not at the ICP4 and LAT promoter regions (*P* = 0.5174 and *P* > 0.9999, respectively) (Fig. 3F).

In summary, the results of ChIP and ChART-PCR analyses are consistent with the HSV-1 genomes in the 2D cultures being associated with heterochromatin during the latent phase of infection, which is consistent with the RT-qPCR data demonstrating repression of lytic genes in a manner similar to that seen in *in vivo* latency models.

### Modeling HSV-1 infections in three-dimensional cultures.

**FIG 4:**
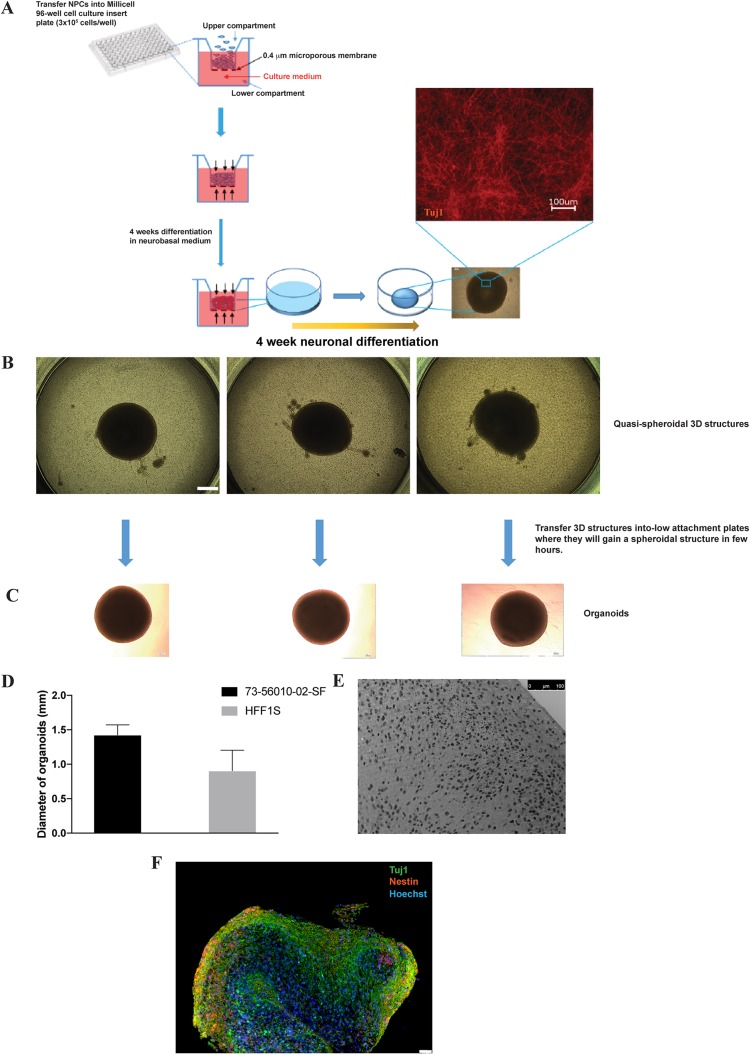
Generation of brain organoids in Millicell-96 cell culture insert plates. (A) Schematic representation of the differentiation procedure. NPCs were seeded at a density of 3 × 10^5^ cells/well on 96-transwell plates. Cells were differentiated for 3 to 4 weeks, as described in the Materials and Methods section. (B) During this period, NPCs self-assembled and organized into multiple layers, forming quasispheroidal structures. (C) These 3D structures were then collected, transferred individually into low-attachment 24-well plates (where, over the course of hours, they assumed a roughly spherical shape), and cultured for an extended period of time. (D) The average size of the organoids after 8 weeks of NPC differentiation is depicted. (E) Hematoxylin and eosin staining of a 10-week-old organoid. (F) Wide-field micrograph of a formalin-fixed, paraffin-embedded section coimmunostained with Tuj1 and nestin. Nuclei were counterstained with Hoechst 33342. Bars, 500 mm (B) and 50 mm (F).

The consistent and efficient chemically induced HSV-1 reactivation in latently infected two-dimensional (2D) cultures of CNS neurons conflicts with the findings of *in vivo* studies from animal models, which cumulatively suggest that there is inefficient reactivation of HSV-1 in the CNS. We reasoned that three-dimensional cultures, such as brain organoids that resemble brain tissue at both the anatomical level and the developmental level, may represent a physiologically relevant *in vitro* system to model HSV-1 infections of CNS (26). We therefore developed an hiPSC-based 3D brain organoid model that combines a highly efficient protocol to generate NPCs from hiPSCs (17) which then relies on the ability of NPCs to self-assemble and self-organize into 3D structures (27). A synopsis of the generation of a 3D neuronal culture is depicted in Fig. 4A. In this work flow, NPCs are generated from hiPSCs as previously described (17) and seeded at a density of 3 × 10^5^ cells/well in Millicell 96-well cell culture insert plates, as detailed in the Materials and Methods section. During the differentiation process, NPCs spontaneously assemble and organize into a multilayered single spheroid-shaped 3D structure (Fig. 4B). The resulting assemblies, when transferred individually to low-attachment 24-well plates, continue to undergo gross morphological changes that result in their becoming more spherical over the course of hours (Fig. 4C). The 3D structures in this study are referred to as organoids and are capable of being cultured in 24-well low-attachment plates for more than 20 weeks. The diameter of the organoids generated from 73-56010-02-SF hiPSCs after 10 weeks of differentiation was remarkably consistent (1.42 ± 0.147 mm, *n* = 22; Fig. 4D). This consistency in size was also observed among organoids generated from HFF1S NPCs (1.130 ± 0.151 mm, *n* = 10; Fig. 4D), which were somewhat smaller than the ones generated from O2SF NPCs. We examined the morphological and neurochemical characteristics of organoids by light and high-resolution confocal microscopy, which revealed an elaborate, three-dimensional loosely laminated arrangement of NPCs and neurons (Fig. 4E and F). The organoid cytoarchitecture (hematoxylin-eosin histochemistry) is complex, with cells exhibiting the morphological appearance of pyramidal neurons localizing to central regions of the organoid and NPC-like cells concentrating toward the periphery of the organoid (Fig. 4E). The 3D structure of the organoid appears to be supported by the production and secretion of extracellular matrix (ECM) molecules chondroitin sulfate proteoglycans (types A and C) (28) (see Fig. 6C), as well as tenascin C (29) (see Fig. 6D) and collagen (see Fig. 6E).

**FIG 5:**
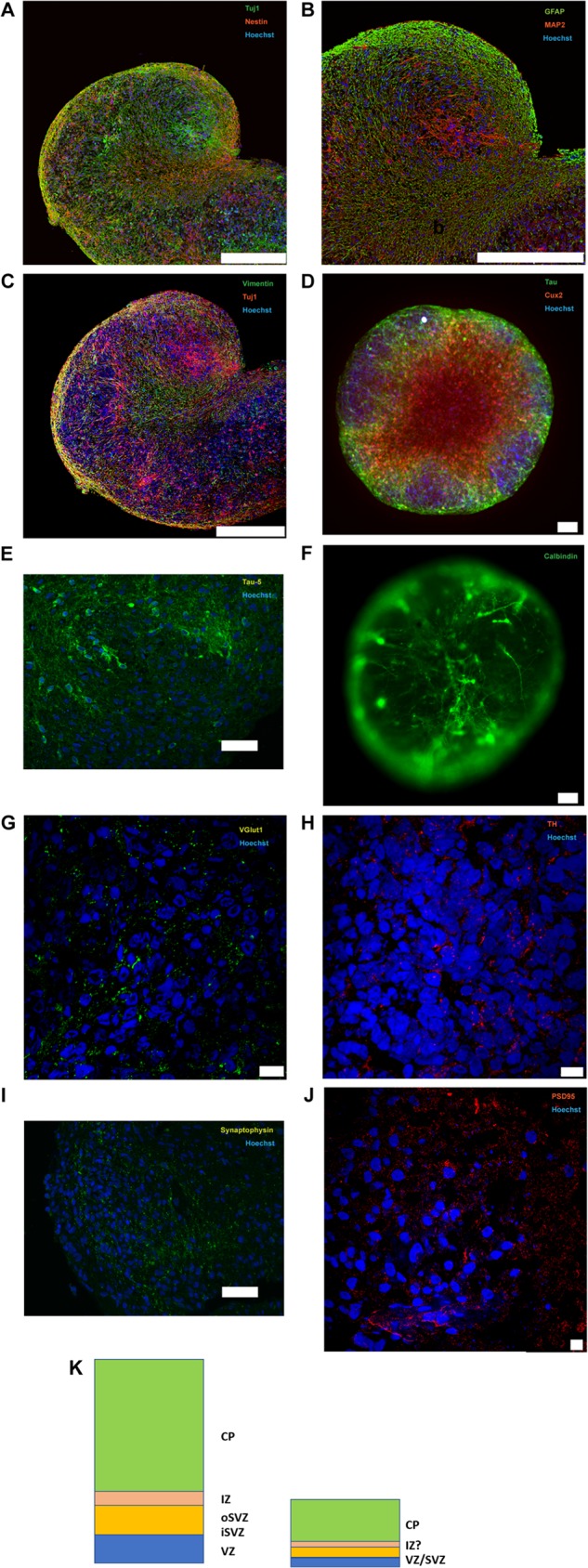
Characterization of brain organoids. (A to J) Immunostaining of formalin-fixed, paraffin-embedded sections, paraformaldehyde-fixed frozen sections of organoids, or paraformaldehyde-fixed whole-brain organoids with Tuj1/nestin (A), GFAP/MAP2 (B), vimentin/Tuj1 (C), Tau (HT7)/Cux2 (D), Tau-5 (E), calbindin (F), VGlut1 (G), TH (H), synaptophysin (I), and PSD95 (J). (D and F) Immunostaining of whole-brain organoids. Nuclei were counterstained with Hoechst 33342. Bars, 250 μm (A to C), 10 μm (G, H, and J), 50 μm (E and I), and 100 μm (D and F). (K) Comparison of the organization of a developing human brain with the organoids generated in this study. VZ, ventricular zone; iSVZ, inner subventricular zone; oSVZ, outer subventricular zone; IZ, intermediate zone; CP, cortical plate.

The phenotype of organoid cells in the outer layer differs from that in the central region of the structure in a manner that is very similar to the laminar organization of the CNS (Fig. 5). The outer region (50 to 150 μm from the edge range) of the organoid contained cells expressing markers typical of brain ventricular and subventricular zones, including nestin (Fig. 5A), GFAP (glial fibrillary acidic protein) (Fig. 5B), and vimentin (Fig. 5C) as well as the homeodomain transcription factor Cux2 (Fig. 5D). Cux2 exerts important regulatory functions in the formation of upper cortical layer neurons (30) and granule cell layer neurons of the dentate gyrus (31).

**FIG 6:**
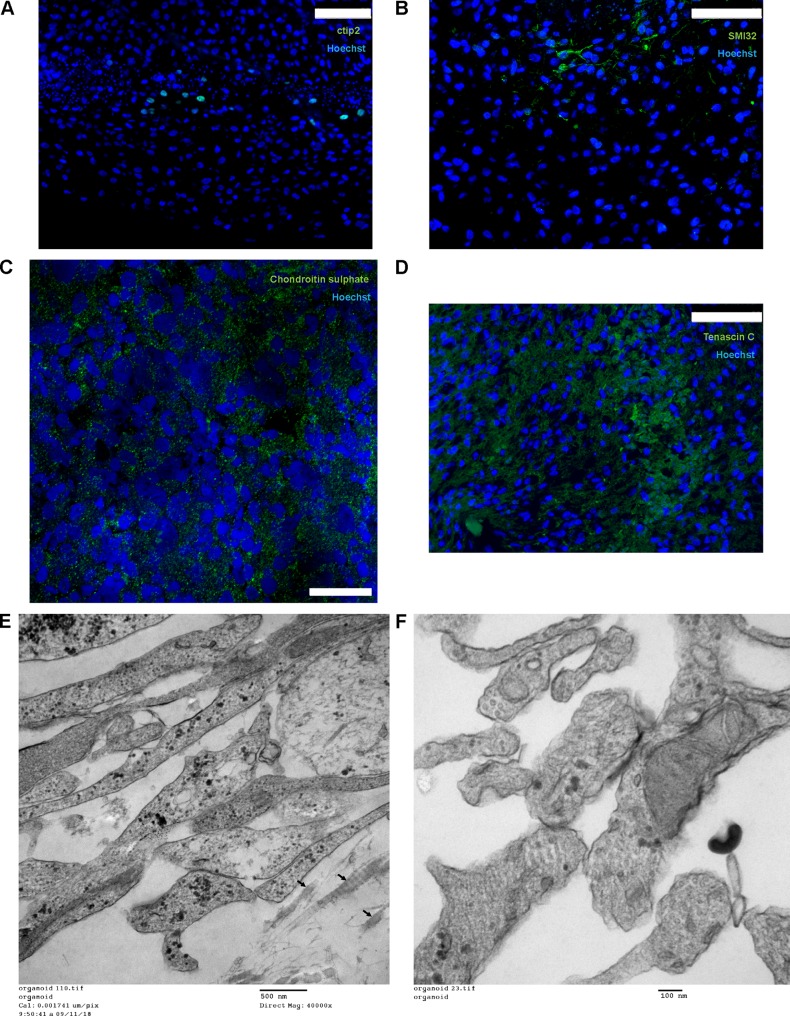
Characterization of brain organoids. (A to D) Immunostaining of formalin-fixed, paraffin-embedded sections of organoids with Ctip2 (A), SMI-32 (B), chondroitin sulfate (C), and tenascin C (D). Bars, 75 mm (A, B, and D) and 20 mm (C). (E and F) Transmission electron microscopy photographs depicting collagen (arrows) as a component of the extracellular matrix (E) and putative synapses (F) in 5-month-old organoids. The organoids were processed for electron microscopy as previously described (63). Nickel grids were examined on a JEOL 1011 transmission electron microscope with a side mount AMT 2k digital camera (Advanced Microscopy Techniques, Danvers, MA).

Immunohistochemical analysis using antibodies generated against Tuj1, MAP2, or Tau showed that these neuronal markers localized to cells located in more central regions of the organoid (Fig. 5A to E). Cells expressing neuronal markers also expressed molecules found in neocortical and hippocampal neurons, including the calcium binding protein Calbindin (32, 33) (Fig. 5F); Ctip2, a transcription factor expressed in the cortical layer 5 (Fig. 6A) and in granule cells that populate the dentate gyrus (34); and SMI-32, a marker of nonphosphorylated neurofilaments (heavy chain) (Fig. 6B) (35–37). Immunoreactivity for the glutamatergic marker VGlut1 was detected (Fig. 5G). Furthermore, a robust differentiation of NPCs in tyrosine hydroxylase (TH)-positive neurons was observed (Fig. 5H). Collectively, these results indicate that under our culture conditions NPCs differentiate into CNS cells exhibiting feature of both cortical and hippocampal cells. Transmission electron microscopy analysis of 5-month-old organoids showed low levels of apoptotic cell death (data not shown).

Processes originating from cells with neuron-like phenotypes and morphology were examined using confocal immunofluorescence as well as electron microscopy. The expression of the presynaptic marker synaptophysin and the postsynaptic marker PSD95 (38, 39) was detected (Fig. 5I and J), suggestive of synaptic specializations. Ultrastructural analyses showed numerous appositions, between 100 and 300 nm long, between membranes. Many of these appositions were formed by neuronal processes resembling neurites. These contained numerous densely packed clear, small vesicles clustered near the contact area. The contact area appeared electron dense in the membrane opposed to the neurite-like processes relative to nonapposed membranes. Mitochondria were detected within the putative neurites and their contacts (Fig. 6F).

### Lytic HSV-1 infection of 3D organoid structures.

**FIG 7:**
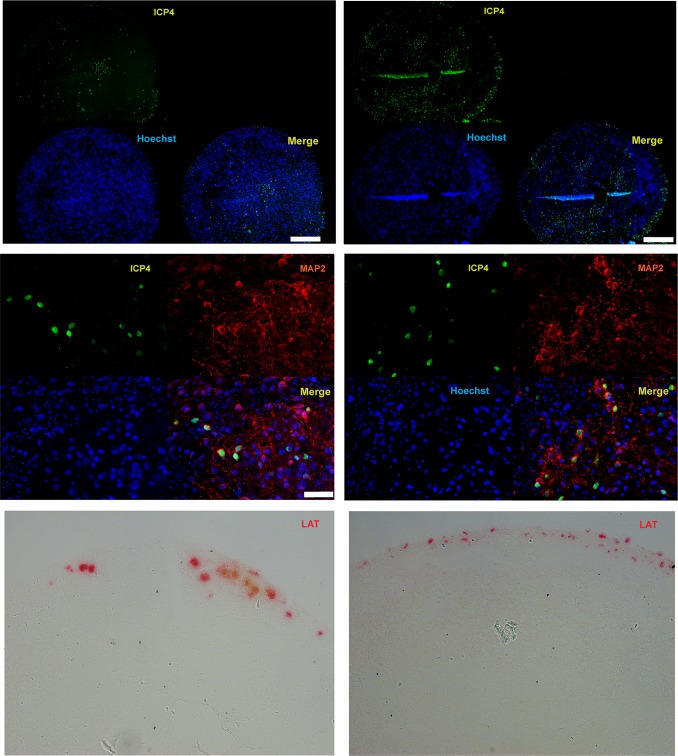
Analysis of ICP4 and LAT expression in HSV-1 acutely infected 10-week-old organoids at 48 h postinfection. (Top) Immunostaining of two paraffin-embedded sections of the same organoid infected with the HSV-1 immediate early gene ICP4. (Middle) Coimmunostaining of a HSV-1-infected organoid with ICP4 and the neuronal marker MAP2. (Bottom) Analysis of LAT expression by RNAscope *in situ* hybridization analysis. Nuclei were counterstained with Hoechst 33342. Bars, 250 μm (top) and 50 μm (middle).

To assess the ability of HSV-1 to infect different cell layers composing the inner and outer portions of the organoids, these 3D structures were incubated with HSV-1 strain KOS, using 1,500 PFU/organoid, by directly adding the virus to the organoids in a small volume of medium (see the Materials and Methods section). The rationale for this inoculum dose was based on estimation of the number of cells present on the surface of the organoids that would be accessible to the infecting virions. Based on imaging cell counts, we estimated that there are approximately 24,000 cells per organoid (data not shown). Based on this estimate and the diameter of the structures, we estimated that there would be approximately 2,000 cells on the circumference of the organoid. Therefore, a dose of 1,500 PFU/organoid seemed like a reasonable dose that should permit a majority of the cells on the surface to be infected with the virus. Viral infection efficiency was assessed by immunohistochemistry after fixing the organoids at 48 hpi in formalin and embedding them in paraffin. Immunohistochemistry analysis of paraffin sections showed cells expressing the HSV-1 immediate early gene ICP4 throughout the organoids (i.e., in the superficial cell layers as well as the more central, neuron-rich areas) (Fig. 7). This result suggests that 3D cell structures can be efficiently infected by HSV-1.

**FIG 8:**
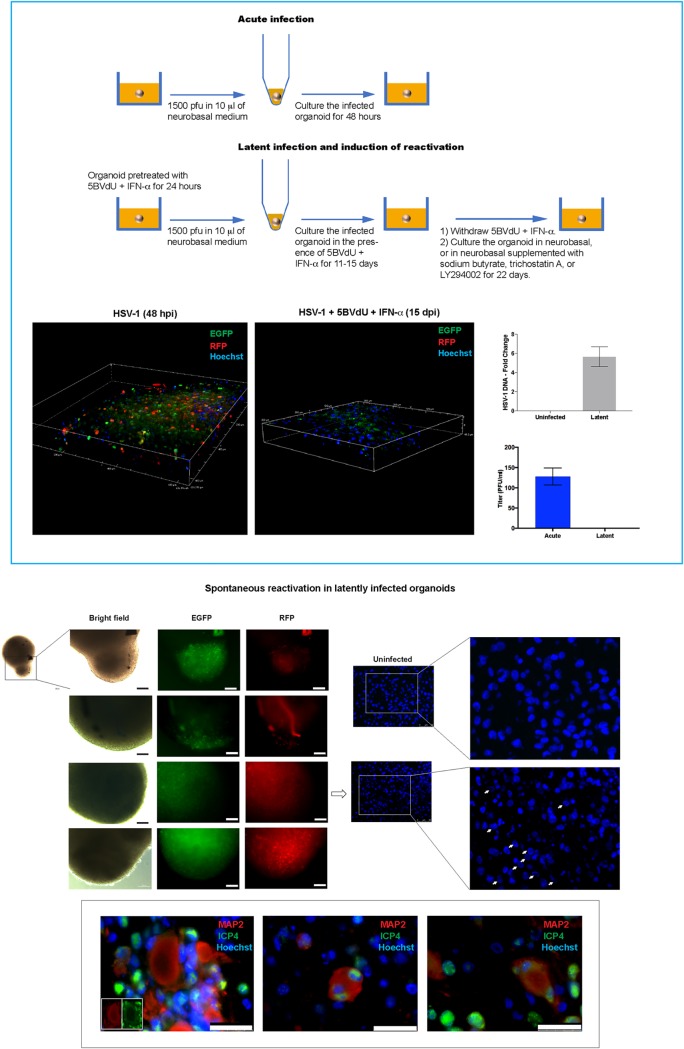
Reduced likelihood of spontaneous or induced reactivation of HSV-1 in brain organoids. Organoids were infected with an HSV-1 construct expressing the reporter genes EGFP and RFP under the control of the HSV-1 promoters ICP0 and gC, respectively, in the absence or the presence of the antivirals 5BVdU and IFN-α (to establish latency). Under the latency condition, the expression of the reporter genes was not observed in 19 out of 23 infected organoids. Paraffin-embedded sections of these latently infected organoids were used to detect viral DNA and analyze the distribution of the host chromatin in infected nuclei. The presence of viral particles in the culture medium of acutely and latently infected organoids was analyzed. (Top box) (Top) Flow chart illustrating acute, latent infection and chemical treatment to induce reactivation. (Bottom left and middle) Confocal imaging of a few layers of organoids infected with HSV-1 in the absence or the presence of 5BVdU and IFN-α; (bottom right) (i) detection of HSV-1 DNA in paraffin-embedded sections of latently infected organoids by quantitative PCR (qPCR) (top) and (ii) viral titer in the supernatants from the acutely and latently infected cultures, measured using a plaque assay on Vero cells (bottom). (Middle) Microphotographs depicting spontaneous HSV-1 reactivation in four latently infected organoids (left) and Hoechst staining of paraffin-embedded sections of an uninfected organoid and an organoid where HSV-1 reactivation was observed (middle and right, where the panels on the right are enlargements of the boxed regions in the panels in the middle). Arrows indicate nuclei showing host chromatin reorganization, which is typically observed in HSV-1-infected cells. (Bottom) Detection of cell-cell fusion during HSV-1 reactivation in organoids. (Insets) Details enlarged in the middle and right panels. Nuclei were counterstained with Hoechst. Bars, 100 μm (middle left) and 25 μm (bottom).

Coimmunostaining showed the expression of ICP4 in the nuclei of MAP2-positive neurons (Fig. 7), consistent with the results from 2D neuronal cultures (Fig. 2) that CNS neurons are permissive to HSV-1. Plaque assays on Vero cell monolayers showed that infectious viral particles were detectable in the supernatant of acutely infected organoids but not in the supernatant of latently infected organoids (Fig. 8). RNAscope *in situ* hybridization analysis revealed LAT expression in a proportion of cells nearest the 3D cell structure surface at 48 hpi (Fig. 7). Considering that the cells composing the outer layers of the organoids are the first ones to be infected, it is conceivable that LAT-positive cells may be detected in deeper layers of the organoids at later time points.

### Latent HSV-1 infection of organoids.

To investigate whether HSV-1 latency can be established in 3D hiPSC-derived neuronal cultures, brain organoids were infected with the HSV-1 strain KOS expressing enhanced green fluorescent protein (EGFP) and red fluorescent protein (RFP) under the control of immediate early and late gene promoters, respectively (1,500 PFU/organoid), and then grown in the presence or the absence of the antivirals 5BVdU and IFN-α (Fig. 8) (see the Materials and Methods section). The expression of the fluorescent reporters EGFP and RFP was observed in the organoids infected in the absence of antiviral drugs (Fig. 8) at 48 hpi, as expected, but in the presence of 5BVdU and IFN-α, only 4 of 23 organoid cultures showed EGFP-positive (EGFP^+^) and RFP-positive (RFP^+^) cells after 8 and 11 days (Fig. 8). Twelve of the remaining 19 infected organoids were then cultured in the absence of 5BVdU and IFN-α to evaluate spontaneous reactivation or exposed to either the histone deacetylase inhibitor sodium butyrate (NaB; 5 mM), the activator trichostatin A (TSA; 1 mM), or the phosphatidylinositol 3-kinase inhibitor (PI3Ki) LY294002 (20 μM) to induce viral reactivation (Fig. 8). Organoids were monitored on a daily basis, using fluorescence microscopy, for spontaneous or induced reactivation. Surprisingly, no EGFP^+^ and RFP^+^ cells were detected after 22 days in any cultures (data not shown). The organoids were then formalin fixed and paraffinized. To investigate the nuclear distribution of the host chromatin, paraffin sections were stained with Hoechst 33342. The displacement of the host chromatin toward the nuclear periphery, which occurs during acute HSV-1 infection (19), was not observed under any of the aforementioned conditions (data not shown). These data suggest that HSV-1 does not efficiently reactivate under conditions known to reactivate from peripheral neuron cultures. The difficulty of HSV-1 reactivation from latency in brain organoids does, however, parallel the low efficiency of HSV-1 reactivation in the CNS (as opposed to peripheral ganglia) seen in animal models (40, 41).

### Analysis of HSV-1 spontaneous reactivation in brain organoids.

As described above, HSV-1 spontaneous reactivation was observed in 4 out of 23 organoids infected and treated with the antivirals 5BVdU and IFN-α: 2 at day 8 postinfection and 2 at day 11 postinfection. One of the two 3D structures displaying viral reactivation at day 8 was composed of two conjoined organoids, and EGFP^+^ and RFP^+^ cells were observed in only one of these two units (Fig. 8). Fluorescent microscopy analysis indicated no spread of the viral infection from one unit to another during the following 72 h; in the second organoid, the EGFP^+^ and RFP^+^ cells were confined in a small area of one organoid (Fig. 8), and the distribution of the fluorescent cells did not change over time. These results indicate that the HSV-1 reactivation observed at 8 dpi could have been abortive. The EGFP^+^ and RFP^+^ cells were distributed across both organoids in which reactivation was observed at 11 days p.i. (Fig. 8). The immunocytochemistry analysis of the neuronal marker MAP2 and the viral protein ICP4 performed on formalin-fixed paraffin sections of these two organoids showed that viral reactivation was accompanied by (i) an abnormal subcellular distribution of MAP2, (ii) a loss of neuronal processes, and (iii) cell-cell fusion (Fig. 8).

## DISCUSSION

Animal models have been invaluable for probing viral life cycles and the mechanisms underlying their pathogenesis in the CNS, although the findings obtained with all such models must be taken with caution because the events seen in the animal may not mimic the events occurring in the natural host. Although HSV-1 can infect and establish latency in a wide range of animal species, it is important to appreciate that HSV-1 coevolved only with humans. As such, it is likely that there are mechanisms of regulating latency that are likely quite specific for human neurons. There are several known differences: for example, experimental reactivation in mice takes considerable effort and is quite inefficient, while reactivation in rabbits is spontaneous but does not require functional TK, as it seems to in humans. We are now at a stage where human *in vitro* model systems have rapidly developed and become available from advancements in stem cell-based technologies. The organ relevance of these stem cell-based *in vitro* systems has increased substantially with the generation of the three-dimensional (3D) cultures called brain organoids, which were used here and which recapitulate aspects of the structural and architectural organization of a developing brain (26). An example of their use has been in understanding Zika virus neuropathogenesis (42–44).

In this study, we first assessed hiPSC-derived 2D neuronal cultures to model aspects of HSV-1 lytic, latent, and reactivated infections. Of the multiple staining and characterization approaches used, the whole-cell patch-clamp experiments are critical as they authenticate functional synaptic contacts in hiPSC-derived neurons (Fig. 1). We provide evidence that (i) hiPSC-derived CNS neurons are permissive to HSV-1 full replication and (ii) HSV-1 can establish latency in hiPSC-derived CNS neurons, in which the genome remains with little lytic promoter activity. We showed expression of the immediate early gene ICP4 in the nuclei of MAP2-positive neurons in culture (Fig. 2), further supporting our work and that of others (11, 27, 45–53) that established the permissiveness of CNS and CNS-like cultured neurons to HSV-1. A previous detailed characterization of HSV-1-infected rodent CNS organotypic cultures by electron microscopy clearly demonstrated the presence of nucleocapsids in 66% of neurons at 24 h postinfection (49).

We have shown that HSV-1 can establish latency in hiPSC-derived neurons, exhibiting some of the main landmarks of HSV-1 latency (11). As seen elsewhere, latency requires a protocol to reduce lytic infections from spreading in the cultures; this protocol uses a combination of the antivirals 5BVdU and alpha interferon (20). Spontaneous reactivation is not detected after the withdrawal of 5BVdU and IFN-α for 5 days but is consistently observed after exposure to sodium butyrate, a histone deacetylase inhibitor (11). FISH analysis of HSV-1 genomes revealed a change in the signal distribution upon HDAC inhibition (Fig. 2). To understand the basis of lytic repression in this model, histone marks were studied; we observed increased levels of H3K27me3 and Bmi1 and reduced levels of H3K4me3 at specific viral promoter regions in latently infected neurons treated with 5BVdU and IFN-α (Fig. 3). This suggests that the culture conditions drive viral chromatin into a heterochromatic state. RT-qPCR analysis showed a drastic reduction in the LAT expression level between acutely and latently infected neurons (Fig. 2). We believe that our results could reflect the lower levels of LAT expression that are observed in CNS neurons *in vivo* than in neurons of the sensory ganglia (54). Interestingly, ChIP analysis showed an enrichment of the transcriptional corepressor KAP1 in the ICP4 promoter region. KAP1 participates in the STAT3-mediated maintenance of latency of Kaposi's sarcoma-associated herpesvirus (KSHV) (24). This regulatory role of STAT3 in the maintenance of latency is carried out, at least in part, by inhibiting the expression of the KSHV immediate early gene RTA (55), which plays a pivotal role in the switch from latency to the lytic viral phase. KAP1 has previously been found to be associated with the HSV-1 genome (56). The enrichment of KAP1 on the ICP4 promoter in our HSV-1 latency model poses the question of whether this corepressor plays a role in the maintenance of the HSV-1 latent state.

While useful, our 2D cultures of CNS neurons did not recapitulate the difficulty of HSV-1 reactivation in the CNS of animal models. Thus, we investigated a 3D culture model. The 3D culture systems appear to mimic aspects of brain architecture and allow complex cell-cell communication, cell-cell interaction, and cell-extracellular matrix interaction. Indeed, the last few years have witnessed growing interest in 3D cultures called organoids to model host-pathogen interactions. Brain organoids have given a great impetus to understanding Zika virus neuropathogenesis (16, 42, 44). We generated scaffold-free brain organoids in 96-transwell plates. Dual immunohistochemical analysis with ICP4 and MAP2 of brain organoids acutely infected for 48 h provided additional evidence about the permissiveness of hiPSC-derived CNS neurons to HSV-1. LAT expression could be detected by RNAscope *in situ* hybridization analysis in cells located in the outer layers of organoids infected in the absence of the antivirals 5BVdU and IFN-α, consistent with the observation in the mouse that the number of cells expressing HSV-1 LAT increases as latency is established (57).

Organoids infected with HSV-1 expressing the reporters EGFP and RFP under the control of the ICP0 and gC viral promoters, respectively, showed that, in the presence of 5BVdU and IFN-α, spontaneous reactivation was observed in approximately 17% of the organoids between days 8 and 11 after infection and antiviral removal. HSV-1 reactivation caused dramatic neuronal morphological changes, consisting of degeneration of neuronal processes and cell-cell fusion, with a consequent generation of neuronal syncytia (Fig. 8). Interestingly, treatment of latently infected organoids with reactivation stimuli, including NaB, TSA, or PI3Ki, for 22 days did not lead to any indicators of viral reactivation or renewed expression of the fluorescent reporters. Thus, the weight of evidence suggests that HSV-1 reactivation occurs infrequently in our 3D model. Indeed, it was very difficult to induce reactivation in the majority of infected organoids. Studies with larger sample sizes are needed to verify these conclusions. In addition, we do not yet know whether the numbers of HSV-1 genomes per cell or the chromatin structure in the 3D cultures is similar to or different from that in the 2D cultures and the role that this could play in reactivation efficiency. The difficulties in inducing HSV-1 reactivation in brain organoids are in stark contrast to the consistent viral reactivation in our latently infected 2D cultures exposed to NaB (11). Differences in cell-cell interactions, cell-cell communications, cell-extracellular matrix interactions, and host gene expression profile have been observed between 2D and 3D cultures of the same cell type (58, 59). These differences may model the difficulties of stimulating HSV-1 to reactivate in the brain organoids and highlight the need to investigate host-pathogen interactions in biological systems that recapitulate the *in vivo* tissue architecture.

### Conclusions.

In summary, we used human hiPSC-derived 2D and 3D cell culture systems to model HSV-1 infection of human neurons. Starting from the invaluable knowledge about HSV-1 neuropathogenesis gained using animal models, hiPSC-based 3D organoids offer an unprecedented opportunity to model HSV-1–neuron interactions in a human context. The differentiation procedure described in this study to generate organoids is scaffold free and relatively cost-effective. Furthermore, the generation of organoids showing consistent diameters could enable the development of drug screening platforms. While these cultures are not a replacement for animal models to study systemic aspects of infections and the role that the adaptive immune system plays, they present themselves as in invaluable tool to study more complex cell and tissue architecture *in vitro* and will allow biochemical and molecular analyses that would be difficult or impossible to perform *in vivo* to be performed.

## MATERIALS AND METHODS

### Cell lines.

Vero cells (CCL-81; ATCC) were maintained in Eagle’s minimum essential medium (EMEM) supplemented with 10% fetal bovine serum (FBS; HyClone) and 5% antibiotic-antimycotic (HyClone). The human iPSC lines 73-56010-02-SF and HFF1S employed in this study were generated from fibroblasts derived from skin biopsy samples which were collected from a healthy individual by the use of a 4-mm full-thickness punch biopsy while the individual was under local anesthesia. The hiPSCs were established at the National Institute of Mental Health (NIMH) Center for Collaborative Studies of Mental Disorders-funded Rutgers University Cell and DNA Repository (RUCDR; http://www.rucdr.org/mental-health). All cells were cultured under standard conditions (37°C, 5% CO_2_, and 100% humidity).

### Generation of 2D neuronal cultures.

The NPCs were derived from hiPSCs as previously described (17) and cultured in neurobasal medium supplemented with 2% B27 and brain-derived neurotrophic factor (BDNF) at 10 ng/ml for 6 weeks. Half of the culture medium was changed every other day.

### Generation of 3D neuronal cultures (brain organoids).

The NPCs were generated from hiPSCs as previously described (17), seeded at a density of 3 × 10^5^ cells/well in Millicell 96-well cell culture insert plates (Millipore), and cultured in neurobasal medium supplemented with 2% B27, BDNF at 10 ng/ml, CHIR9901 at 3 μM, forskolin at 10 μM, dorsomorphin at 1 μM, 50 U/ml penicillin G, and 50 mg/ml streptomycin. After 4 days, CHIR990, forskolin, and dorsomorphin were withdrawn and the cells were cultured for an additional 4 weeks. Half of the medium in the upper and lower compartments was changed every other day. During the process of differentiation, NPCs self-assembled into round, quasispheroidal structures. In the initial stages of self-assembly, the culture pulled away from the walls of the Millicell wells and condensed, forming a 3D reticular structure consisting of a ring crossed by a spindle-like agglomerations of cells, roughly resembling a wheel with spokes. Over time these spokes merged with the outer ring structure, forming a crescent shape and then a toroid, the center of which filled in to form quasispheroidal structures. These structures were then detached by applying a mild pressure with a P-1000 pipette using wide bore tips and transferred singularly into low-attachment 24-well plates, where they were cultured for up to 20 months. Half of the culture medium in the upper and lower compartments was changed every other day.

### Electrophysiological recordings.

Whole-cell recordings were performed at room temperature on hiPSC-derived neurons plated on 35-mm culture dishes. The extracellular solution contained 140 mM NaCl, 2.8 mM KCl, 1 mM CaCl_2_, 0.01 mM EDTA, and 10 mM HEPES, with the pH adjusted to 7.2 with NaOH and the osmolarity adjusted to 283 to 284 mmol/kg. Borosilicate patch pipettes were pulled to obtain tip resistances of 5 to 9 MΩ with the P-1000 pipette puller (Sutter Instrument, Novato, CA, USA). The pipette solution contained 130 mM K-gluconate, 10 mM NaCl, 1 mM CaCl_2_, 1 mM EGTA, 10 mM HEPES, 2 mM ATP-Na_2_, and 2 mM MgCl_2_, with the pH adjusted to 7.2 with KOH and the osmolarity adjusted to 280 mmol/kg. Patch pipettes were connected to a Multiclamp 700B (Axon CNS; Molecular Devices, Sunnyvale, CA, USA) amplifier interfaced with an Axon Digidata 1500 system (Axon Instrument; Molecular Devices, Sunnyvale, CA, USA). Currents were sampled at 10 kHz and low-pass filtered at 5 kHz. AxoScope (version 10.4; Molecular Devices, Sunnyvale, CA, USA) and pClamp (version 10.4; Molecular Devices, Sunnyvale, CA, USA) software was used to acquire and analyze the data.

Membrane currents were recorded using 400-ms depolarizing voltage steps from − 100 mV to 20 mV starting from the holding potential of −85 mV. Action potentials were evoked in current-clamp mode, injecting 500-ms depolarizing steps of current from −100 pA to 80 pA starting from the holding current of −60 pA.

### Viral infections.

The following HSV-1 strains were employed in this study: KOS (VR-1493; ATCC), a KOS-based recombinant construct incorporating enhanced green fluorescent protein (EGFP) and monomeric red fluorescent protein (RFP) as reporters whose gene expression is driven by the viral promoters ICP0 and glycoprotein C, respectively (60), and 17*syn*+ (obtained by David C. Bloom).

### (i) Infection of 2D neuronal cultures.

For lytic infections, cell-free virus was adsorbed onto monolayer hiPSC-derived neurons at a multiplicity of infection (MOI) of 0.3. At 2 h after the infection, the inocula were then removed and the cells were washed twice with Dulbecco modified Eagle medium (DMEM)–Ham’s F-12 medium and cultured with neurobasal medium for 24 h. For latent infections, cells were preincubated with 5BVdU and IFN-α. After 24 h, cells were infected as described above and cultured with neurobasal medium in the presence of 5BVdU and IFN-α for 7 days. The culture medium was changed every other day.

### (ii) Infection of brain organoids.

Brain organoids were infected as follow. Organoids cultured in low-attachment 24-well plates were transferred singularly in 1.5-ml Eppendorf tubes and washed with 500 μl of DMEM–Ham’s F-12 medium. The medium was then discarded and 10 μl of neurobasal medium with or without 5BVdU and IFN-α containing 1,500 PFU of HSV-1 was added. After perforating the cap of the Eppendorf tubes using a 20-gause sterile needle, the organoids were cultured in an incubator under standard conditions (5% CO_2_, 37°C, 100% humidity). To inhibit viral replication, the organoids were pretreated with 5BVdU and IFN-α for 24 h. At 2 h after the infection, the inoculum was removed and the organoids were washed twice with 500 μl of DMEM–Ham’s F12 medium and cultured in neurobasal medium in the presence or the absence of 5BVdU and IFN-α in low-attachment 24-well plates.

### FISH.

The distribution pattern of the HSV-1 genomes in the infected nuclei was analyzed by performing three-dimensional fluorescence *in situ* hybridization (3D-FISH) as described by Cremer et al. (61). The HSV-1 genome was used as a probe. On average, 50 nuclei from each cellular preparation were scanned. Digital images were generated using the Leica LAS AF software 3D visualization module. Hybridization signals were subjected to uniform thresholding to demarcate the signals.

### Immunofluorescence.

The 2D neuronal cultures were fixed with 4% paraformaldehyde and permeabilized with 0.2% Triton X-100 before immunostaining.

The paraffin-embedded slices of organoids were prepared as follows. The organoids were rinsed in phosphate-buffered saline (PBS) and fixed by immersing them in at least 10 volumes of 10% formalin overnight at 4°C. The organoids were again rinsed and then embedded in blocks of low-melting-point agarose. The agarose blocks were embedded in paraffin wax following a standard protocol for formalin-fixed tissue and then sectioned to 5 μm for subsequent staining. Before staining, paraffin sections were incubated at 60°C; dewaxed in xylene; hydrated in absolute ethanol, 95% ethanol, and 70% ethanol; and rinsed in pure water. Antigen unmasking was performed by exposing the paraffin sections to antigen retrieval Citra solution (BioGenex) at 95°C. Paraffin sections were incubated with SuperBlock blocking buffer (Thermo Scientific) before immunostaining. Paraffin sections were also stained with hematoxylin and eosin reagents.

To prepare frozen sections, the organoids were transferred into cryomolds (Tissue-Tek cryomold intermediate). After adsorbing traces of culture medium, the organoids were frozen in cryomolds (Tissue-Tek cryomold intermediate) and embedded into OCT medium at −22°C. Sections of 5 μm were prepared by use of a cryostat (Micron HM350; Thermo Fisher Scientific). Frozen sections were stored at −80°C until needed. Before staining, frozen sections were air dried, fixed with 4% paraformaldehyde for 20 min, and incubated with SuperBlock blocking buffer for 1 h at room temperature.

Whole organoids were fixed with 4% paraformaldehyde for 4 h. Before staining, fixed organoids were permeabilized with 0.2% Triton X-100 overnight at 4°C.

Samples were incubated with primary antibodies overnight at 4°C. The primary antibodies used were mouse monoclonal anti-β-tubulin III antibody (conjugated clone TUJ1; 1:100 dilution; catalog no. NL1195V; R&D Systems), mouse monoclonal anti-MAP2 (1:500 dilution; catalog no. AB5622; Millipore), chicken polyclonal anti-GFAP (1:500 dilution; catalog no. ab4674; Abcam), mouse monoclonal anti-VGlut1 (1:500 dilution; catalog no. ab150347; Abcam), rabbit polyclonal anticalbindin (1:400 dilution; catalog no. ab11426; Abcam), rabbit polyclonal antivimentin (1:500 dilution; catalog no. ab45939; Abcam), rabbit monoclonal antinestin (1:1,000 dilution; catalog no. ABD69; Millipore), mouse polyclonal anti-PSD95 (1:400 dilution; catalog no. ab18258; Abcam), mouse polyclonal anti-TH (1:1,000 dilution; Abcam), rabbit monoclonal antisynaptophysin (1:500 dilution; catalog no. 611880; BD Biosciences), mouse monoclonal anti-Tau-5 (1:500 dilution; catalog no. AHB0042; Innovative Research), rabbit polyclonal anti-Cux2 (1:200 dilution; catalog no. ab130395; Abcam), mouse monoclonal anti-Tau (1:500 dilution; catalog no. MN100D; Invitrogen), and mouse monoclonal anti HSV-1 ICP4 (dilution 1:200; catalog no. ab6514; Abcam). Alexa Fluor 488 goat anti-rabbit immunoglobulin secondary antibody (1:300 dilution; catalog no. A-11008; Thermo Fisher Scientific), Alexa Fluor 488 goat anti-mouse immunoglobulin secondary antibody (1:300 dilution; catalog no. A-10680; Thermo Fisher Scientific), Alexa Fluor 594 goat anti-rabbit immunoglobulin secondary antibody (1:300 dilution; catalog no. A-11012; Thermo Fisher Scientific), Alexa Fluor 594 goat anti-mouse immunoglobulin secondary antibody (1:300 dilution; catalog no. A-11005; Thermo Fisher Scientific), Alexa Fluor 488 goat anti-chicken immunoglobulin secondary antibody (1:300 dilution; catalog no. A-11039; Thermo Fisher Scientific), and Alexa Fluor 488 goat anti-rat immunoglobulin (1:300 dilution; catalog no. 711-545-152; Jackson ImmunoResearch Labs) were used for detection. A Nikon A1 confocal microscope was used to collect a virtual stack of optical sections taken from the sample. A 20× dry objective and a 60× oil objective were used for acquisition with a Galvano scan unit, with sequential scanning for proximal wavelengths at a 0.5-μm optical section thickness and a 1,024-by-1,024 resolution, using conservative laser power settings and GaAsp detectors on the 488-nm and 560-nm laser lines.

### RT-PCR.

Double-stranded cDNAs were prepared from 6-week-old hiPSC-derived neurons through random primed reverse transcription using SuperScript II reverse transcriptase (Invitrogen). The following primers were used: hGluRB Q/R F (5′-TTTAGCCTATGAGATCTGGATGTGC-3′), hGluRB Q/R R (5′-GTGTAGGAGGAGATTATGATCAGG-3′), hGluR5 F (5′-GCTTGGGAGTCAGCTGTGTA-3′), hGluR5 R (5′-ATGGGGGATTCCATTCTCTC-3′), hGluR6 F (5′-ACCTTGCAGTTGCTCCACTGG-3′), and hGluR6 R (5′-ACCTTGCAGTTGCTCCACTGG-3′).

Endogenous control gene probes were specific to beta-actin (ACTB) and spanned exons 2 and 3 (catalog no. 4310881E; Applied Biosystems).

For actin, thermal cycling conditions were 95°C for 10 min, followed by 30 cycles at 94°C for 30 s, 56°C for 30 s, and 72°C for 1 min and then a final extension at 72°C for 7 min. For glutamatergic receptor genes GluRB, GluR5, and GluR6, thermal cycling conditions were 95°C for 10 min, followed by 10 cycles at 94°C for 15 s, 68°C to 59°C for 30 s (the temperature was decreased by 1°C at each cycle), and 72°C for 30 s and then 35 cycles at 95°C for 15 s, 60°C for 30 s, and 72°C for 30 s and a final extension at 72°C for 7 min.

### RT-qPCR.

RNA was extracted from mock-infected or HSV-1 infected 6-week-old hiPSC-derived neurons with the TRIzol reagent and spin column purification (catalog no. 12183555; Thermo Fisher Scientific). RNA samples were treated with Turbo DNase for 30 min at 37°C, followed by incubation with DNase inactivation reagent for 5 min at room temperature (Turbo DNA-free kit; catalog no. AM1907; Thermo Fisher Scientific). Reverse transcription was performed with 20-μl reaction mixtures and a RETROscript kit (catalog no. AM1710; Thermo Fisher Scientific) with either random decamers for GAPDH, ICP4, TK, and gC or gene-specific primers for analysis of LAT expression. RT reaction mixtures were prepared per the manufacturer’s recommendation, and the RT reactions with the lytic transcripts were performed by incubating 1 μg of RNA with Moloney murine leukemia virus (MMLV) reverse transcriptase for 60 min at 42°C, followed by inactivation at 92°C for 10 min. For gene-specific RT, a primer spanning the HSV-1 LAT intron (5′-GTG GTC GGA CGG GTA AGT AA-3′) was used, and reactions were performed as follows: 5× annealing buffer (100 mM Tris-HCl, pH 8.3, 50 mM KCl) was added to the reaction mixture at a final 1× concentration in a mixture containing 20 μM primer and 20 U RNasin, and the reaction mixture was incubated for 10 min at 37°C. One microgram of RNA was next added, the mixture was heated for 3 min at 80°C, and then primer annealing was performed for 45 min at 50°C. cDNA was generated by adding MMLV reverse transcriptase, deoxynucleoside triphosphates, and RT buffer in a final reaction volume of 25 μl and incubating at 50°C for 45 min. The reverse transcriptase enzyme was inactivated by incubation at 92°C for 10 min, and qPCR was performed using the following Applied Biosystems TaqMan assay: human GAPDH (catalog no. 4331182; assay identifier, s02786624_g1). The following primers were used: LAT intron F (5′-CGCCCCAGAGGCTAAGG-3′), LAT intron R (5′-GGGCTGGTGTGCTGTAACA-3′), LAT intron probe (5′-CCACGCCACTCGCG-3′), ICP4 F (5′-CACGGGCCGCTTCAC-3′), ICP4 R (5′-GCGATAGCGCGCGTAGA-3′), ICP4 probe (5′-CCGACGCGACCTCC-3′), TK F (5′-CACGCTACTGCGGGTTTATATAGAC-3′), TK R (5′-GGCTCGGGTACGTAGACGATAT-3′), TK probe (5′-CACCACGCAACTGC-3′), gC F (5′-CCTCCACGCCCAAAAGC-3′), gC R (5′-GGTGGTGTTGTTCTTGGGTTTG-3′), and gC probe (5′-CCCCACGTCCACCCC-3′).

### ChIP.

hiPSC-neurons generated in Matrigel-coated 6-well plates (1.5 × 10^6^ to 2 × 10^6^ cells/well) were infected at multiplicities of infection (MOI) of 0.3 in the presence of the antivirals (*E*)-5-(2-bromovinyl)-2′-deoxyuridine (5BVdU; 30 μM) and alpha interferon (IFN-α; 125 U/ml) for 7 days to establish latent infection. Cells were also acutely infected at the same MOI for 24 h. Chromatin was prepared from acutely and latently infected cells. Following chromatin cross-linking and cell lysis, the chromatin was sonicated to generate fragments with sizes of 100 to 500 bp. Chromatin immunoprecipitation (ChIP) assays were performed using a ChromataChIP kit (Novus Biological) according to the manufacturer’s instructions with anti-histone H3K27me3 antibody, anti-histone H3K4 antibody, anti-Bmi1 polycomb protein, anti-KAP1 antibody, or anti-HP1 antibody. Two micrograms of the aforementioned antibodies was used for each ChIP assay. Negative controls were samples to which no antibody was added. Input DNA (nonimmunoprecipitated), immunoprecipitated DNA, and DNA from the no-antibody condition were recovered from acutely and latently infected cultures and were analyzed by TaqMan real-time PCR in triplicate using primer pairs specific for the following HSV-1 genes: ICP4, ICP27, and LAT regions. The following primers and probes were used: primer GAPDHpF (5′-AGACCTGGGCTGGGACT-3′), primer GAPDHpR (5′-GAACAGGAGGAGCAGAGAGC-3′), probe GAPDHp (5′-6FAM-AAATTGAGCCCGCAGCCTCC-TAMRA-3′, where 6FAM is 6-carboxyfluorescein and TAMRA is 6-carboxytetramethylrhodamine), primer RHOpF (5′-TGACCTCAGGCTTCCTCCTA-3′), primer RHOpR (5′-ATCAGCATCTGGGAGATTGG-3′), probe RHO (5′-6FAM-ATTAGGCCCTCAGTTTCTGCAGCG-TAMRA-3′), primer ICP4pF (5′-GACGTAGCACGGTAGGTCAC-3′), primer ICP4pR (5′-CTTTTTCCCACCCAAGCAT-3′), probe ICP4p (5′-6FAM-CCGTCGACGCGGAACTAGCG-TAMRA-3′), primer ICP27pF (5′-CGGCCTGACAGAGCTGTATT-3′), primer ICP27pR (5′-CCGAGAGGATGATGGAACAG-3′), probe ICP27p (5′-6FAM-AAGGGGCTGTCGGGCGTC-TAMRA-3′), primer gCpF (5′-TGTGTGATGATTTCGCCATAA-3′), primer gCpR (5’ATGGGGGTGTGAGTTCGAT-3′), probe gC (5′-FAM-CACTACCGAGGGCGCTTGGT-3′), primer LATpF (5′-CAATAACAACCCCAACGGAAAGC-3′), primer LATpR (5′-TCCACTTCCCGTCCTTCCAT-3′), and probe LATp (5′-6FAM-TCCCCTCGGTTGTTCC-TAMRA-3′).

The following qPCR conditions were used: (i) for host genes *GAPDH* and *RHO*, 95°C for 3 min, followed by 45 cycles of 95°C for 15 s and 60°C for 30 s and a finish with 72°C for 30 s, and (ii) for viral genes ICP4, ICP27, gC, and LAT, 95°C for 12 min, followed by 45 cycles of 95°C for 15 s and 55°C for 1 min.

### Data analysis.

ChIP experiments were carried out with three biological samples for each condition. ChIP-qPCR was performed in triplicate, and the data were normalized using the percent input method (62). The threshold cycle (*C_T_*) values from 1% input were normalized to 100% by subtracting the log_2_ value of 100 (6.64). The fold difference between immunoprecipitated (ChIP) samples and the normalized input for each sample was calculated as follows: percent input = 2^[(*CT* input 6.64) − (*CT* ChIP)]^ × 100. The no-antibody signal was subtracted from the ChIP signal for each target gene. The enrichment of H3K27me3, H3K4, Bmi1, and KAP1 at the promoter region of the indicated HSV-1 genes in latently infected neuronal cultures compared to their levels in acutely infected cultures was assessed using Student's *t* test.

### ChART-PCR.

Approximately 5 × 10^6^ cells were acutely and latently infected. Nuclei from both conditions were isolated using a Nuclei EZ Prep kit (Sigma) according to the manufacturer’s instructions. Aliquots of nuclei were digested with increasing amounts of MCN (0.5 U, 1 U, 3 U, and 5 U) at 39°C for 20 min. The nuclease activity was stopped by adding 5 mM EGTA, and DNA was isolated using standard procedures. A control without MCN was included. Aliquots of each of these partial MCN digestions were run on a 1.5% agarose gel to examine the extent of the digestion. Five nanograms of undigested and partially MCN-digested DNA from each condition was used to perform TaqMan real-time PCR. The set of primers and the reaction conditions were identical to those described above.

One-way analysis of variance (ANOVA) was performed on three infection groups for four viral gene promoter Δ*C_q_* values separately. A *post hoc* Tukey test was performed on groups that showed significant differences across the mean Δ*C_q_* values. ANOVA and *post hoc* Tukey tests were performed in GraphPad Prism (version 7) software.

### qPCR assays to detect viral DNA in brain organoids.

DNAs from paraffin-embedded tissues were extracted using a TaKaRa Dexpat kit according to the manufacturer’s instructions. The qPCR conditions were the same as those described above.

Institutional Review Board (IRB) approval for research involving human subjects was obtained from the University of Pittsburgh Institutional IRB under IRB number PRO09080146.
